# Health Risk Perception, Consumption Intention, and Willingness to Pay for Pig Products Obtained by Immunocastration

**DOI:** 10.3390/ani10091548

**Published:** 2020-09-01

**Authors:** Jorgelina Di Pasquale, Yari Vecchio, Giovanna Martelli, Luca Sardi, Felice Adinolfi, Eleonora Nannoni

**Affiliations:** 1 Faculty of Veterinary Medicine, University of Teramo, 64100 Piano D’Accio, Teramo, Italy; jdipasquale@unite.it; 2 Department of Veterinary Medical Sciences, University of Bologna, Via Tolara di Sopra 50, 40064 Ozzano Emilia (Bologna), Italy; giovanna.martelli@unibo.it (G.M.); luca.sardi@unibo.it (L.S.); felice.adinolfi@unibo.it (F.A.); eleonora.nannoni2@unibo.it (E.N.)

**Keywords:** animal welfare, consumer behaviour, risk perception, willingness to pay, pig, castration, immunocastration, vaccination, alternative methods, consumption

## Abstract

**Simple Summary:**

Public awareness of farm animal welfare has gradually increased. It is well-known that some routine procedures in pig farming are detrimental to animal welfare, including surgical castration, especially without anaesthesia and/or analgesia. Some alternative techniques that offer welfare advantages over surgical castration can be implemented—immunocastration is among them. However, producers fear that consumers may be frightened by this technique and therefore they delay changes in the production system. This work aimed to understand whether Italian consumers perceive a health risk from the immunocastration technique and whether this perception is connected with their willingness to consume and to pay for products derived from immunocastrated animals. The results show that, within the Italian population, there are different levels of perceived health risk and that, as the perception of risk increases, the willingness to consume products obtained from immunocastrated animals decreases (and *vice versa*). The health risk perception by consumers also changes the willingness to pay for immunocastrated products. Particular attention must be paid to the information transmitted to the consumer if this technology will be widely adopted in pig husbandry.

**Abstract:**

Surgical castration without the use of anaesthesia and/or analgesia is considered to be detrimental for the welfare of pigs and for this reason its abandonment is advocated. Immunocastration is a more welfare-friendly alternative method; however, stakeholders in the pork sector fear consumer rejection due to perceived safety issues of immunocastrated meat. This work aimed to analyse whether Italian consumers perceive a health risk arising from the use of this technique and, if so, how the perceived risk may influence the purchase choices and the willingness to pay for products derived from immunocastrated animals. To achieve this objective, a survey was carried out on a representative sample of the Italian population. The results highlight that consumers perceive different levels of risk related to the use of immunocastration and that this influences purchasing behaviour and willingness to pay. Moreover, it should be noted that the willingness to pay is also influenced by certain demographic factors, since this is positively associated with younger respondents with lower incomes and less knowledge of farming systems, who live in rural areas and have a greater sensitivity to animal welfare. Given the concerns expressed by consumers, particular attention must be paid to the information transmitted if this technology will be widely implemented in pig husbandry.

## 1. Introduction

Several reasons determined the adoption of castration in pig breeding [1]. In addition to reasons related to animal management, such as reducing aggression and sexual behavior [2], the most relevant motivation was the need to reduce the boar taint in the meat of entire male pigs [3]. In these animals, androstenone and skatole accumulate in fat tissues and create a smell and a taste commonly referred to as “boar taint”, which are not appreciated by consumers [4,5]. However, not all consumers have the same sensitivity to the two molecules. Skatole is perceived by almost all consumers (99%) [6], while androstenone is perceived by about half of them (40–50%) [7,8,9,10]. Moreover, some studies show that there is a difference in sensitivity related to gender, with women being more sensitive to the presence of androstenone than men [7,9,11,12,13].

The technique most widely used in Europe to avoid boar taint is castration without the use of anaesthesia and analgesia [14]. This kind of castration can be carried out in accordance with the legislation within seven days of life [15]. The increased sensitivity of European consumers towards the welfare of farm animals has led to a desire to abandon this practice and to adopt alternative techniques, as reported in the European Declaration on alternatives to the surgical castration of pigs [16].

Some countries, such as Spain and the United Kingdom, have chosen to breed entire males, other countries have adopted surgical castration with the use of anaesthesia and analgesia, or have implemented the use of immunocastration. In other contexts, the operators of the pig supply chain try to reduce boar taint risk through nutritional strategies and genetic selection [14,17,18,19].

Each of these techniques has pros and cons that must be taken into account within each production system [18,19,20,21,22]. In addition to the economic and business management factors related to the introduction of a new production technique, one additional aspect to be taken into consideration is consumer acceptability. Several studies have investigated consumers’ ability to accept boar taint [7,9,12,23,24] and the possibility of masking this smell through different food preparation methods [25,26,27,28]. Other studies have focused their attention on consumers’ acceptability of immunocastration applied in order to improve animal welfare [29,30,31,32,33,34,35,36], but few studies have analysed the perception of risk tied to the application of immunocastration by consumers and the relative willingness to accept this technique, aspects which are fundamental to its implementation [37,38,39,40]. Assuming that few consumers are aware that male piglets are surgically castrated without anaesthesia or analgesia, as highlighted in a recent review by Mancini et al. [41], and that few consumers are familiar with the immunocastration technique, it is important to understand their attitude once they are made aware of its possible use, so as not to run into an ancestral rejection of the unknown. According to Claude Fischler’s theory [42], men lead their controversial existence between two poles, that of the fear of contamination (neophobia) and that of the tension towards change and diversification (neophilia). Obviously, since the knowledge of foods derives from experience, humans expose themselves to risk at every intake of a new food. Many studies analyzed the perception of risk by consumers. This branch of literature has its roots in the perceived risk theory, initially used in marketing to understand consumer behaviour when making purchasing decisions with imperfect information ([43,44,45,46,47,48,49,50,51,52] among others). As regards products of animal origin, other studies addressed the risks associated with the presence of chemical and biological contaminants [53,54,55,56], or the use of biotechnologies [57,58], as well as meat consumption and focus, for example, on the consumption of chicken meat [59,60,61,62], red meat [63,64,65,66], and pork [67,68,69,70].

The multiple food scandals of the last twenty years (BSE Bovine Spongiform Encephalopathy, Avian Influenza, *Escherichia coli* O104: H4; Melamine milk) have sensitized the public opinion to reject the use of many new technologies in the food sector, such as GMOs (Genetically Modified Organisms) [57,58]. These attitudes alarm producers, who fear consumers may reject the use of the immunocastration vaccine [19,71,72,73].

Therefore, a good understanding of consumers’ perceptions and attitudes towards innovations in food products and a good use of innovation are crucial for the successful introduction of novelty [74,75].

For this reason, the purpose of this paper is twofold: on the one hand, to investigate the level of health-related risk perceived by consumers for what concerns immunocastration and, on the other hand, to verify the existence of a relationship between perceived health risk and propensity to consume and between perceived risk and willingness to pay (WTP). In particular, our analysis aimed to examine how the perception of risk affects purchasing behaviour by determining a higher or lower propensity to consume products obtained through this technique and to investigate how risk perception affects personal preferences in purchasing products obtained through the use of alternative production techniques. Furthermore, it also intends to identify the main variables that influence WTP and how WTP varies according to purchase preferences.

Our research hypothesis is based on the existence of a significant relationship between the perception of risk and the willingness to consume products obtained from immunocastrated animals. Furthermore, we hypothesize different levels of risk perception within the population, resulting in different propensities to consume, preferences for alternative production techniques, and WTP.

Our research questions are therefore expressed as follows: 1. What is the relationship between risk perception for the use of immunocastration and the propensity to consume the products thus obtained? 2. What is the relationship between risk perception and the preferences of consumption for products obtained through alternative production techniques? 3. What factors can influence the WTP for products obtained through immunocastration?

This document aims to contribute to the abundant literature ([41,76,77] among others) on the WTP for products with higher animal welfare standards than current ones, trying to fill the knowledge gap on the effect that the perception of risk for immunocastration has on personal preferences.

## 2. Materials and Methods

### 2.1. Research Approach and Sampling

Between December 2018 and January 2019, a representative sample of the Italian population of pork consumers was surveyed. Participation quotas were identified by gender, age (older than 18 years) and geographical area (North-West, North-East, Centre, South, Islands) [78]. The questionnaire was designed by the authors based on a review of the literature available on immunocastration. Subsequently, a pre-test was carried out with 20 volunteers to verify that questions were understandable to readers and that there was no risk of misinterpretation. The questionnaire was slightly modified based on volunteers’ feedback. These twenty respondents were not included in the final survey. Afterward, a pilot survey was carried out on a sample of 126 interviewees.

In the questionnaire used for the study, consumers were asked to read a short paragraph containing information about immunocastration, which was previously tested by experts and consumers to ensure it was neutral. Any word that could voluntarily elicit emotions was carefully eliminated from the text, such as “piglets”, “pain”, or word such as “much” or “little”. Likewise, any concept that might reveal a preference by the authors for one technique over another was avoided (the paragraph is provided in the supplementary material Table S1). This step aimed to increase the consumers’ knowledge of the investigation topic. The survey was carried out by a specialized agency, with CAWI (Computer Assisted Web Interview) methodology. The response rate was 73%, and 3.8% of responses were screened-out because respondents were non-consumers of pork. The final sample consisted of 969 Italian respondents. The questions used for the analysis are presented below in the specific sections of the manuscript.

### 2.2. Statistical Analysis

All analyses were conducted with SPSS V.25.0 (IBM Corp., Armonk, NY, USA, Released 2017)

#### 2.2.1. Correlation Analysis to Verify Which Relationship Exists between the Perception of Risk for the Use of Immunocastration and the Propensity to Consume the Products Thus Obtained

In order to assess the relationships between the level of risk perceived by consumers in relation to immunocastration and the propensity to consume products derived from immunocastrated animals, a correlation between two variables was tested: the first one capable of gathering information on the willingness to consume products from immunocastrated animals defined as variable 1 “willingness to consume”, and the second one suitable for collecting information relating to the perception of risk, defined as variable 2 “risk perception”.

“Willingness to consume” is a continuous variable, for which data were collected by means of an open question (asking to express a level on a scale from 0 to 100), submitted to the consumer in the following way: “Assuming that the abandonment of surgical castration and the adoption of immunocastration would improve pig welfare, to what extent would you be willing to consume products obtained through the use of immunocastration? Please rate your score on a 0 to 100 scale”.

“Risk perception” is, similar to “willingness to consume”, a continuous variable, for which data were collected by means of an open question which was submitted to the consumer in the following way “Please indicate (express it as a percentage) to what extent do you think that immunocastration might carry some risks (even if still unknown) for consumers’ health _______________ %”.

The correlation between these two variables should indicate whether the increase in the perception of risk relating to foods obtained from animals subjected to immunocastration decreases (or not) the willingness to consume these products. The Pearson correlation coefficient was used to carry out this analysis.

#### 2.2.2. ANOVA Test to Verify the Relationship between the Perception of Risk and the Preferences of Consumption for Products Obtained through Alternative Breeding Techniques

In order to verify how the perception of risk can influence consumers’ purchasing choices, the analysis carried out several steps:

Firstly, respondents had to indicate their purchasing preference among the alternative methods to avoid boar taint. Or rather, they were offered the opportunity to choose one among five alternative methodologies found in the literature (Table 1). In this way the sample self-selected into five groups.

Subsequently, two one-way ANOVA tests were carried out on the five groups thus obtained. The first one-way ANOVA test was performed to verify whether the level of willingness to consume products obtained from immunocastration was different and statistically significant among the groups of consumers who declared different preferences. The second one-way ANOVA test was performed to check whether the level of the perceived risk for products obtained from immunocastrated pigs was different among the five groups.

The first test used variable 1 “willingness to consume” as its dependent variable, while in the second one, we used variable 2 “risk perception” as the dependent variable.

In order to validate the quality of the analysis, robustness tests were conducted. Based on the characteristics of the variables tested, the Brown and Forsythe [79] and Welch [80] tests were chosen. Moreover, pairwise comparisons were carried out by using Duncan [81] and Scheffe [82] post hoc tests to confirm the absence of significant differences between all possible pairs of averages. Statistical significance was set at *p* < 0.05 for all tests.

#### 2.2.3. WTP-Logit Regression to Verify Which Factors Can Influence the Willingness to Pay for Products Obtained through Immunocastration and Assessment of How the Willingness to Pay Varies according to Purchase Preferences

In order to identify the characteristics that determine the highest probability that an individual is willing to pay a premium price for a food obtained through the use of immunocastration, a logistic regression was carried out on the entire sample representative of the Italian population. Subsequently, within each self-selected group based on the preference for alternative breeding techniques, we assessed the average value that each group of individuals is willing to pay for immunocastrated product.

The logistic regression analysis is mainly used to deal with the choice of a binary scenario [83].

In fact, the independent part is represented by a binary variable to which is linked a random variable Y_ì_ with a Bernoullian distribution. It is used above all for its predictive function, with which it is possible to estimate the probability that in an independent data event, *n* regressors will occur. In the interpretation of the result, the beta parameter, which illustrates the negative or positive effects on the dependent variable by the regressors, must be estimated. In our case we use Wald’s test to estimate the existence of a statistically significant relationship. In addition, the results of the maximum probability (ML) algorithm and the R2 values of Cox and Snell and Nagerkelke will be commented on to estimate the goodness of the model [84,85].

In our case, we have selected as a dichotomous dependent variable the willingness to pay a premium price or not for products obtained through the technique of immunocastration. As independent variables, we included all the characteristics identified in the literature as determining factors in the decision to buy products with higher standards of animal welfare.

The variables used are the following (Table 2).

The logistic model was conducted using the stepwise technique. This technique consists of a method of adaptation of the regression models, in which the predictors are automatically chosen through the use of a predetermined criterion. In our case, we used a stepwise forward technique using Wald’s statistic.

In order to identify the average price increase consumers would pay for products derived from immunocastration, the following question was asked: “Which premium price, expressed as a percentage, would you be willing to pay for immunocastrated pork? Please rate your score on a 0 to 100 scale___________________%”.

Subsequently, the average premium price that consumers are willing to pay for products obtained through immunocastration was calculated both for the entire surveyed sample and for each group defined based on the preferred alternative techniques, regardless of their consumption preference.

## 3. Results

This section is divided by subheadings, in order to provide a more precise description of the experimental results.

### 3.1. Results of Correlation Analysis to Verify What Type of Relationship Exists between the Perception of Risk for the Use of Immunocastration and the Willingness to Consume the Products Thus Obtained

The correlation analysis based on Pearson’s correlation coefficient between the variables 1 “willingness to consume” and 2 “risk perception” for consumers’ health indicated a statistically significant correlation, with an inversely proportional weak ratio (Table 3), that is, as the perception of the risk for the health of the consumer increases, the willingness to consume the products obtained through immunocastration decreases.

### 3.2. Results of ANOVA Test to Verify the Relationship between the Perception of Risk and the Consumption Preferences for Products Obtained through Alternative Breeding Techniques

Concerning the methods used to avoid boar taint, consumers have indicated their preference by choosing among the four methods proposed in the literature and the methodology widely in use at present (surgical castration without the use of anaesthetics). The different methodologies have different levels of preference among consumers. The least preferred technique within the population is surgical castration without the use of anaesthesia/analgesia, i.e., the most widespread, which was chosen by only 9.2% of consumers. This methodology is followed by the choice to breed animals genetically selected for their low risk of developing boar taint, which was preferred by 15.9% of consumers. The remaining three techniques, surgical castration with the use of anaesthesia/analgesia, breeding of entire animals, and immunocastration, are preferred by a fifth of consumers for the first two and by a third of consumers for the third (Table 4).

One-way ANOVA highlighted a different propensity to consume immunocastrated meat among groups, as well as a statistically significant difference in the perception of risk (Table 5). That is, the consumers who indicated their preference for traditional surgical castration are also those who are less willing to consume food products derived from immunocastrated pigs, followed by those who would accept boar taint in meat if entire animals were raised and those who would prefer castration with the use of anaesthesia/analgesia. Consumers who prefer the technique of immunocastration and perceive the lowest risk are, of course, more willing to consume these products. A higher risk is perceived by consumers who would prefer traditional castration.

Similarly, pairwise comparisons show that consumers preferring meat from immunocastrated animals have statistically higher willingness to consume these products and statistically lower perceived risk levels compared to all the other groups of consumers.

On average, consumers indicated a perceived level of risk below 35, which is relatively low since it refers to a 0–100 scale, and a relatively high (almost 55%) willingness to consume products derived from immunocastrated animals (Table 5).

The existence of statistically significant differences between the different groups considered is demonstrated by a highly significant *p*-Value (less than 0.001). Therefore, the five groups can be considered different with regard to their propensity to consume and propensity to risk. The most important differences are between group 1 (traditional surgical castration) and group 5 (immunocastration) (Table 5) both in terms of propensity to consume and risk perception, which can be expected given the negative correlation of the variables tested. This leads us to accept the null hypothesis of equal propensity to consume and perception of risk. The two robustness tests of Welch and Brown-Forsythe (Table 6) were carried out and confirmed the possibility of accepting the null hypothesis since the significance of the test is less than 0.05.

Subsequently, post hoc tests (Duncan and Scheffe; Supplementary Materials Table S3) were performed, which confirmed the presence of statistically significant differences and therefore the presence of heterogeneous subsets for alpha = 0.05.

### 3.3. Results of Logit Regression to Verify Which Factors Can Influence the Willingness to Pay for Products Obtained through Immunocastration

Assuming the null hypothesis to be true, the model correctly predicts 26.1% of the observations, but does not consider the presence of the independent variables. Instead, if the model is tested by including the independent variables, the correct percentage increases to 73.9%, thus confirming the alternative one as the best hypothesis. The results of the model are shown in Table 7.

If we set the error tolerated at 5% (*p*-value = 0.05) with six degrees of freedom, the value of the statistics of the distribution table is 12.59. In our case, the Chi square assumes the value of 283.958; therefore, we can reject the null hypothesis, because the value of the chi square obtained from our data turns out to be superior to that shown in Table 5. This, if we set the error at 5%, therefore represents a good measure of likelihood for our test. A high value of the Cox and Snell R Square and Nagelkerke R Square indices is a measure of the model’s robustness. As shown in Table 5, the Cox and Snell R Square and Nagelkerke R Square values are 0.254 and 0.339, respectively. The value of the likelihood-2 logarithm for the search model is 1059.361. Hair et al. [58] suggest that the lower the probability value of the likelihood logarithm, the better the goodness of the search model.

The independent variables included in the model are statistically significant in identifying the consumers willing to pay a premium price. It turns out that people who are consumers living in rural areas and who are younger and have lower incomes are willing to pay a higher share for “immunocastration” products; these two last measures are significantly correlated according to the Pearson correlation index. Furthermore, individuals who value animal welfare but have less knowledge of the subject and who believe that pigs are raised with acceptable minimum standards tend to spend more.

### 3.4. Results of WTP and Evaluation on How It Varies according to Purchase Preferences

**Figure 1:**
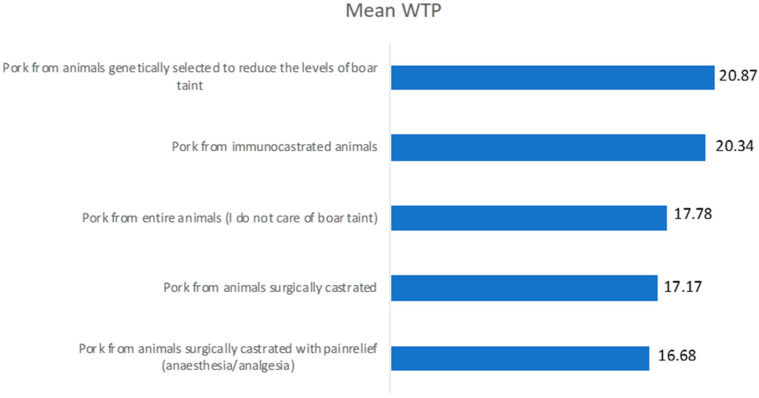
Average percentage increase in WTP (%) for products derived from the immunocastration based on the five different consumer groups.

Once the variables influencing the WTP of Italian consumers were defined, the WTP intensity for products derived from immunocastration of the five previously identified groups was investigated. The results are presented in Figure 1.

The WTP found in all groups is relatively high. It averages around a price increase of 18.6% and ranges from a minimum of +16.68% to a maximum of +20.87%. Consumers who prefer products obtained through the use of surgical castration (with or without the use of anaesthetics) are those who declare a lower WTP. Instead, consumers who accept more easily the use of technology in the food sector, such as genetic selection or the use of a vaccine, are willing to pay a higher premium price.

## 4. Discussion

The propensity to consume products obtained through the use of immunocastration goes hand in hand and in an inversely proportional way to the perception of the risk linked to the use of the technique. In fact, the correlation shown between variable 1 and variable 2 indicates a direct, statistically significant, although not excessively strong link. Consumers who believe that eating food from immunocastrated animals implies no health risk are more willing to consume these products. On the contrary, consumers who think immunocastration can pose a health risk and are somewhat frightened by it are less likely to consume these products. This result confirms first of all that Italian consumers perceive a risk in the application of the immunocastration technique, as evidenced by other works [38,39,60]. However, the level of perceived risk is on average low. Secondly, it shows different levels of risk perception. Even though about 30% of consumers prefer to consume meat from animals that have not been pharmacologically treated (products obtained through the use of surgical castration, 9.2%, and raising entire animals products obtained through the breeding of whole pigs, 20.9%), the percentage of those who would prefer the use of alternative techniques to avoid the boar smell in meat in favour of animal welfare is extremely high (90.8%). This result confirms what has already been widely highlighted in the literature [30,35,39,86,87], that is, the surgical castration without the use of anaesthesia/analgesia has been identified by consumers as a technique not respectful of animal welfare and therefore not desirable, and this is the case also for Italian consumers.

It is possible that immunocastration may not be the only technique to be considered in pig farms, since about one fifth of the interviewed sample would prefer castration with the use of anaesthetics and/or analgesics, another fifth would accept the boar taint in meat; 15% would accept genetic selection of pigs that express this feature to a lower extent. As highlighted by some works [21,41], each of the above-mentioned techniques has managerial and economic constraints that must be taken into due consideration. However, immunocastration remains the technique that received the highest preference (33.6% of the sample).

Consumers who are more inclined to accept innovative techniques, such as the genetic selection of pigs and immunocastration itself, perceive a lower risk in its use and are more willing to consume the products thus obtained. Consumers who are less inclined to consume products derived from immunocastrated pigs are those who perceive a higher risk linked to the use of the technique. These respondents remain anchored to purchasing and consumption routines, and would continue consuming products obtained with surgical castration (with or without anaesthetics/analgesics), or even to accept the boar taint in meat, products which, for their use history can be traced back to the “precautionary principle”. The latter result is in line with the literature, which indicates a greater acceptability of innovation in the food sector when it is configured as incremental innovation, i.e., when the innovation is gradually implemented or when only one attribute of the product is modified [88,89].

The level of perceived risk also has an effect on WTP. The average WTP for products obtained by immunocastration is 18.6%. Nonetheless, consumers who chose products obtained from surgical castration and castration with anaesthesia and/or analgesia, remaining in a “comfort zone”, declared a higher WTP, between 16.68% and 17.78%. Consumers who demonstrate greater openness to new technologies declared a higher WTP, between 20.34% and 20.87%.

This is a declared willingness to pay and is therefore certainly overestimated, but the data indicate a clear trend which confirms some of the hypotheses of our work. The characteristics that determine the willingness to pay a premium price for products obtained through immunocastration are young age and low income. The two variables are related, since often, in Italian society, young people enter the world of work quite late and this determines their economic condition; however, their greater sensitivity towards animal welfare determines a greater propensity to choose foods produced in a more animal-friendly way. The housing environment is also an important determinant of behaviour, and in particular living in a rural context, as is poor knowledge of breeding conditions. This is in contrast to what can be found in the literature, where detachment from rural life determines a greater apprehension for animal welfare [90,91]. The other results are in agreement with the literature in which young age, lack of knowledge of the living conditions of animals, and greater sensitivity towards animal welfare influence WTP [76,77,91,92].

## 5. Conclusions

This work highlighted that Italian consumers perceive a human health-related risk tied to the application of immunocastration. However, there are different levels of risk perception within the population, and these differences are reflected in different types of behaviour and food choices about immunocastration. In other words, the existing and statistically significant relationship between the level of risk perceived by consumers and the propensity to consume these foods determines a different preference for alternative boar taint management strategies. Furthermore, a different level of WTP for products derived from immunocastrated animals could be observed according to different purchasing preferences in relation to the different perceptions of risk. These results indicate that the concerns raised by the producers are not unfounded, as consumers perceived certain risks. However, among the proposed alternative techniques, immunocastration was the preferred technique for more than 30% of the sample, and the overall level of the health-related risk perceived was low (below 35%). If immunocastration will be implemented as a standard production technique, it will be necessary to convey more information to the consumer in order to decrease their perception of the health-related risk and avoid the possibility of a refusal of this production technique due to a lack of knowledge.

## Figures and Tables

**Table 1:** Alternative methodologies found in the literature to avoid boar taint.

Please, Indicate Your Purchasing Preference among the Following Pork Products (Single Choice) ^1,2^	
1	From animals surgically castrated
2	From animals surgically castrated with pain relief (anaesthesia/analgesia)
3	From entire animals genetically selected to reduce the levels of boar taint
4	From immunocastrated animals
5	From entire animals (I do not care about boar taint)

^1^ Note: Raising only female animals has also been proposed in the literature. However, sperm sexing is currently not perfected enough to make this option viable. For this reason, this technique was not offered among the possible choices, but the possibility of including it in “other” was left in case some consumers were aware of it. No consumer chose the “other” option. ^2^ Note: the order of possible choices was randomized.

**Table 2:** List of variables used in logit regression.

Code Variable	Label	Modalities ^1^
Gender	Gender	2
Education	Level of education	6
Income	Income	6
Age	Age of respondents	Free
Home	Do you live in rural or urban area?	2
Aware	Are you aware that male pigs are castrated in their first days of life?	2
Animal Welfare (AW)-importance	Importance given to animal welfare during purchase	7
Pig Welfare	Perceived level of pig welfare on farms	5
Knowledge	Knowledge about AW	5

^1^ Note: The specification of the modalities relating to socio-demographic variables can be viewed in the sample table in Supplementary Materials Table S2.

**Table 3:** Correlation based on Pearson’s correlation coefficient.

Correlations		
	willingness to consume	risk perception
willingness to consume	1	−0.232 ^1^
risk perception	−0.232 ^1^	1

^1^ Correlation is significant at the 0.01 level (2-tailed).

**Table 4:** Consumer preference for one among the alternative methodologies found in the literature to avoid boar taint.

Please, Indicate Your Purchasing Preference Among the Following Pork Products (Single Choice)		
Preferred Alternative Meat Products	Frequency	Percent
From animals surgically castrated	89	9.2%
From entire animals genetically selected to reduce the levels of boar taint	154	15.9%
From animals surgically castrated with pain relief (anaesthesia/analgesia)	198	20.4%
From entire animals (I do not care about boar taint)	202	20.9%
From immunocastrated animals	326	33.6
Total	969	100%

**Table 5:** One-way ANOVA: Willingness to consume based on different alternative methods to avoid boar taint and risk perception for products obtained from immunocastrated pigs.

Statistics	Choice (Means)	Frequency	Willingness to Consume, %	Risk Perception, %
Mean	Pork from animals surgically castrated	89	39.56	43.97
	Pork from entire animals (I do not care about boar taint)	202	44.98	40.11
	Pork from animals surgically castrated with pain relief (anaesthesia/analgesia)	198	49.17	38.07
	Pork from animals genetically selected to reduce the levels of boar taint	154	52.21	40.08
	Pork from immunocastrated animals	326	68.92	22.84
Total		969	54.54	34.23
Anova	F-test		29.276	24.293
	*p*-Value		<0.001	<0.001

**Table 6:** Robust Tests of Equality of Means.

Variable	Test	Statistic ^1^	Sig.
Consume	Welch	30.383	0.000
	Brown-Forsythe	28.733	0.000
Risk	Welch	28.758	0.000
	Brown-Forsythe	21.914	0.000

^1^ Asymptotic F distribution.

**Table 7:** Output and Summary Statistics of the Logit Model.

Variables	B	S.E.	Wald	Sig.	Exp(B)
Age	−0.016	0.005	9.568	0.002	0.984
Income	−0.19	0.065	8.477	0.004	0.827
AW importance	0.096	0.032	9.087	0.003	1.101
Knowledge	−0.216	0.061	12.382	0	0.806
Urban or Rural	−0.521	0.214	5.912	0.015	0.594
Pig welfare	0.177	0.065	7.481	0.006	1.194
−2 Log likelihood	Cox & Snell R Square	Nagelkerke R Square	Chi-Square	Df	Sig.
1059.361	0.254	0.339	283.958	6	0.000

## Supplementary Materials

The following are available online at https://www.mdpi.com/2076-2615/10/9/1548/s1, Table S1: Short, neutral information about immunocastration, Table S2: Socio-demographic variables used in logit regression, Table S3: socio-demographic variables, Output of the post-hoc Scheffe Test (*p* < 0.05).
