# Supplementary material for: Health Risk Perception, Consumption Intention, and Willingness to Pay for Pig Products Obtained by Immunocastration
Source: Animals (Basel). 2020 Sep 1;10(9):1548. doi: 10.3390/ani10091548 (PMC7552121; doi:10.3390/ani10091548)
Supplement: Supplementary file 1 [file animals-10-01548-s001.pdf]

animals

MDPI

Article

Health Risk Perception, Consumption Intention and Willingness to Pay for Pig Products Obtained by Immunocastration

Jorgelina Di Pasquale [1], Yari Vecchio [2,*], Giovanna Martelli [2], Luca Sardi [2], Felice Adinolfi [2] and Eleonora Nannoni [2]

[1] Faculty of Veterinary Medicine, University of Teramo, 64100 Piano D'Accio, Teramo, Italy; jdipasquale@unite.it (J.D.P.)

[2] Department of Veterinary Medical Sciences, University of Bologna, Via Tolara di Sopra 50, 40064 Ozzano Emilia (BO), Italy; giovanna.martelli@unibo.it (G.M.), luca.sardi@unibo.it (L.S), felice.adinolfi@unibo.it (F.A.), eleonora.nannoni2@unibo.it (E.N.)

* Correspondence: yari.vecchio@unibo.it

Received: 07 August 2020; Accepted: 26 August 2020; Published: date

Table S1. Short, neutral information about immunocastration.

**Neutral Information**
In many countries of the world male pigs are castrated. The aim of castration is to avoid the development of objectionable meat odours (the so-called 'boar taint', whose development is due to sexual maturity) and to limit aggression and competition between animals.<br>One of the most frequently used methods (also in Italy) is surgical castration. According to legislation, surgical castration can be carried out without the administration of anaesthetics and/or analgesics when done within the first week of age. After the 7th day of life, castration must be carried out with the administration of anaesthetics and analgesics.<br>In Italy pigs are usually castrated before the 7th day of life.<br>Some alternative castration methods have been proposed. Among these, the most frequently used in countries such as Brazil, New Zealand and Australia is immunocastration. This method consists in the administration of a vaccine which stops the production of sexual hormones, therefore, preventing sexual maturity.

Table S2. Socio-demographic variables used in logit regression.

Gender:

Statistics	Variables	Frequency	Percentage	Percentage Valid	Cumulative percentage
Valid	Male	485	50,0	50,1	50,1
	Female	484	49,9	49,9	100,0
	Total	969	99,9	100,0	
Missing	Sistem	1	,1		
Total	970	100,0			

Education

Statistics	Variables	Frequency	Percentage	Percentage Valid	Cumulative percentage
Valid	Primary school	6	,6	,6	,6
	Middle school	94	9,7	9,7	10,3
	High school	531	54,7	54,8	65,1
	3-year	133	13,7	13,7	78,8

	university degree				
	5-year university degree	205	21,1	21,2	100,0
	Total	969	99,9	100,0	
Missing	Sistem	1	,1		
Total	970	100,0			

Household size

Statistics	Variables	Frequency	Percentage	Percentage Valid	Cumulative percentage
Valid	1	73	7,5	7,5	7,5
	2	233	24,0	24,0	31,6
	3	277	28,6	28,6	60,2
	4	308	31,8	31,8	92,0
	5	68	7,0	7,0	99,0
	6	8	,8	,8	99,8
	7	2	,2	,2	100,0
	Totale	969	99,9	100,0	
Missing	Sistema	1	,1		
Total	970	100,0			

Annual household income:

Statistic	Variables	Frequency	Percentage	Percentage Valid	Cumulative percentage
Valid	-10.000 €	102	10,5	10,5	10,5
	11.000 - 20.000 €	226	23,3	23,3	33,8
	21.000 - 35.000 €	337	34,7	34,8	68,6
	36.000 - 50.000 €	210	21,6	21,7	90,3
	51.000 - 75.000	73	7,5	7,5	97,8
	+ 75.000	21	2,2	2,2	100,0
	Total	969	99,9	100,0	
Missing	Sistem	1	,1		
Total	970	100,0			

Area

Statistic	Variables	Frequency	Percentage	Percentage Valid	Cumulative percentage
Valid	Northwest	248	25,6	25,6	25,6
	North East	186	19,2	19,2	44,8
	Center	183	18,9	18,9	63,7

	South	238	24,5	24,6	88,2
	Island	114	11,8	11,8	100,0
	Total	969	99,9	100,0	
Missing	Sistem	1	,1		
Total	970	100,0			

Urban o Rural Area					
Statistic	Variables	Frequency	Percentage	Percentage Valid	Cumulative percentage
Valid	Urban	847	87,3	87,4	87,4
	Rural	122	12,6	12,6	100,0
	Total	969	99,9	100,0	
Missing	Sistem	1	,1		
Total	970	100,0			

Table S3. Output of post-hoc Scheffe Test (*p*<0.05); * indicates that the mean difference is significant.

Multiple Comparisons						
Dependent Variable				Mean Difference (I-J)	Std. Error	Sig.
Assuming that the abandonment of surgical castration and the adoption of immunocastration would improve pig welfare, at what extent, would you be willing to consume products obtained through the use of immunocastration? Please rate your score on a 0 to 100 scale	Scheffe	Pork from animals surgically castrated	Pork from immunocastrated animals	-29,355*	3,705	0
			Pork from animals surgically castrated with the administration of anesthesia/analgesia	-9,605	3,953	0,207
			Pork from animals genetically selected for their low risk of developing boar taint	-12,652	4,125	0,052
			Pork from entire (non castrated) animals (I do not care of boar taint)	-5,418	3,941	0,756
		Pork from immunocastrated animals	Pork from animals surgically castrated	29,355*	3,705	0
			Pork from animals surgically castrated with the administration of anesthesia/analgesia	19,751*	2,791	0
			Pork from animals genetically selected for their low risk of developing boar taint	16,703*	3,029	0
			Pork from entire (non castrated) animals (I do not care of boar taint)	23,937*	2,774	0
		Pork from animals surgically castrated with the administration of anesthesia/analgesia	Pork from animals surgically castrated	9,605	3,953	0,207
			Pork from immunocastrated animals	-19,751*	2,791	0
			Pork from animals genetically selected for their low risk of developing boar taint	-3,048	3,328	0,933
			Pork from entire (non castrated) animals (I do not care of boar taint)	4,186	3,098	0,768
		Pork from animals genetically selected for their low risk of developing boar taint	Pork from animals surgically castrated	12,652	4,125	0,052
			Pork from immunocastrated animals	-16,703*	3,029	0
			Pork from animals surgically castrated with the administration of anesthesia/analgesia	3,048	3,328	0,933
			Pork from entire (non castrated) animals (I do not care of boar taint)	7,234	3,314	0,313
		Pork from entire (non castrated) animals (I do not care of boar taint)	Pork from animals surgically castrated	5,418	3,941	0,756
			Pork from immunocastrated animals	-23,937*	2,774	0
			Pork from animals surgically castrated with the administration of anesthesia/analgesia	-4,186	3,098	0,768
			Pork from animals genetically selected for their low risk of developing boar taint	-7,234	3,314	0,313
Please indicate (expressing it as a percentage) at what extent do you think that immunocastration might carry some risks (even if still unknown) for consumers' health _______________ %	Scheffe	Pork from animals surgically castrated	Pork from immunocastrated animals	21,132*	3,115	0
			Pork from animals surgically castrated with the administration of anesthesia/analgesia	5,896	3,324	0,534
			Pork from animals genetically selected for their low risk of developing boar taint	3,882	3,469	0,869
			Pork from entire (non castrated) animals (I do not care of boar taint)	3,862	3,314	0,851
		Pork from immunocastrated animals	Pork from animals surgically castrated	-21,132*	3,115	0
			Pork from animals surgically castrated with the administration of anesthesia/analgesia	-15,236*	2,347	0
			Pork from animals genetically selected for their low risk of developing boar taint	-17,250*	2,547	0
			Pork from entire (non castrated) animals (I do not care of boar taint)	-17,270*	2,333	0
		Pork from animals surgically castrated with the administration of anesthesia/analgesia	Pork from animals surgically castrated	-5,896	3,324	0,534
			Pork from immunocastrated animals	15,236*	2,347	0
			Pork from animals genetically selected for their low risk of developing boar taint	-2,014	2,799	0,972
			Pork from entire (non castrated) animals (I do not care of boar taint)	-2,033	2,605	0,962
		Pork from animals genetically selected for their low risk of developing boar taint	Pork from animals surgically castrated	-3,882	3,469	0,869
			Pork from immunocastrated animals	17,250*	2,547	0
			Pork from animals surgically castrated with the administration of anesthesia/analgesia	2,014	2,799	0,972
			Pork from entire (non castrated) animals (I do not care of boar taint)	-0,02	2,787	1
		Pork from entire (non castrated) animals (I do not care of boar taint)	Pork from animals surgically castrated	-3,862	3,314	0,851
			Pork from immunocastrated animals	17,270*	2,333	0
			Pork from animals surgically castrated with the administration of anesthesia/analgesia	2,033	2,605	0,962
			Pork from animals genetically selected for their low risk of developing boar taint	0,02	2,787	1

© 2020 by the authors. Submitted for possible open access publication under the terms and conditions of the Creative Commons Attribution (CC BY) license (http://creativecommons.org/licenses/by/4.0/).
